# Genotyping‐by‐sequencing through transcriptomics: implementation in a range of crop species with varying reproductive habits and ploidy levels

**DOI:** 10.1111/pbi.12835

**Published:** 2017-10-13

**Authors:** M. Michelle Malmberg, Luke W. Pembleton, Rebecca C. Baillie, Michelle C. Drayton, Shimna Sudheesh, Sukhjiwan Kaur, Hiroshi Shinozuka, Preeti Verma, German C. Spangenberg, Hans D. Daetwyler, John W. Forster, Noel O.I. Cogan

**Affiliations:** ^1^ Agriculture Victoria AgriBio Centre for AgriBioscience 5 Ring Road Bundoora Victoria 3083 Australia; ^2^ School of Applied Systems Biology La Trobe University Bundoora Victoria 3086 Australia

**Keywords:** *Brassica napus*, *Lens culinaris*, *Lolium perenne*, *Phalaris aquatic*, RNA

## Abstract

The application of genomics in crops has the ability to significantly improve genetic gain for agriculture. Many marker‐dense tools have been developed, but few have seen broad adoption in plant genomics due to issues of significant variations of genome size, levels of ploidy, single nucleotide polymorphism (SNP) frequency and reproductive habit. When combined with limited breeding activities, small research communities and scant sequence resources, the suitability of popular systems is often suboptimal and routinely fails to effectively balance cost‐effectiveness and sample throughput. Genotyping‐by‐sequencing (GBS) encompasses a range of protocols including resequencing of the transcriptome. This study describes a skim GBS‐transcriptomics (GBS‐t) approach developed to be broadly applicable, cost‐effective and high‐throughput while still assaying a significant number of SNP loci. A range of crop species with differing levels of ploidy and degree of inbreeding/outbreeding were chosen, including perennial ryegrass, a diploid outbreeding forage grass; phalaris, a putative segmental allotetraploid outbreeding forage grass; lentil, a diploid inbreeding grain legume; and canola, an allotetraploid partially outbreeding oilseed. GBS‐t was validated as a simple and largely automated, cost‐effective method which generates sufficient SNPs (from 89 738 to 231 977) with acceptable levels of missing data and even genome coverage from c. 3 million sequence reads per sample. GBS‐t is therefore a broadly applicable system suitable for many crops, offering advantages over other systems. The correct choice of subsequent sequence analysis software is important, and the bioinformatics process should be iterative and tailored to the specific challenges posed by ploidy variation and extent of heterozygosity.

## Introduction

Following the initial conception by Meuwissen *et al*. (2001), genomic selection (GS) has revolutionized the livestock industries such that the majority of dairy cattle bulls are now selected based on genomic estimated breeding values (GEBVs; Pryce and Daetwyler, 2012). The value of the approach has been confirmed in major animal (Garcia‐Ruiz *et al*., 2016; Wolc *et al*., 2015) and plant species (Lin *et al*., 2014; Rutkoski *et al*., 2015). The essence of GS is to extensively phenotype and genotype a reference population, in order to simultaneously predict the effect of all markers. This prediction equation is then used to infer the genetic merit of individuals based only on genotypic profiles. The method assumes causative mutations are in linkage disequilibrium (LD) with at least one sampled genetic marker, and so requires that the genome is saturated with markers, where the density depends on the diversity of the breeding population. GS was initially developed and applied to the few major mammalian and avian livestock species (bovine, ovine, porcine, galline: Groenen *et al*., 2011; Kijas *et al*., 2012; Matukumalli *et al*., 2009; Ramos *et al*., 2009), using high‐density, cost‐effective SNP chip marker systems. In contrast to many plants, these species are all diploid and outbreeding with genomes of broadly similar size (Gregory, 2005), most of which have been compiled as full references (Elsik *et al*., 2009; Groenen *et al*., 2012; Jiang *et al*., 2014). In addition to typically limited genomic resources, crops display a diversity of reproductive habits that can influence the degree of intragenomic heterozygosity, multiple ploidy levels, as well as the distinction between auto‐ and allopolyploidy. Such biological factors have limited commercial opportunities to develop species‐specific SNP chips, as the fabrication costs for small production runs are prohibitive for the number of samples that would be processed.

Plants exhibit the largest variance for genome size of any kingdom (Zonneveld *et al*., 2005). The smallest reported plant genome belongs to the carnivorous *Genlisea margaretae* Hutch, at 63.4 Mbp (Greilhuber *et al*., 2006) while the largest reported eukaryotic genome belongs to the subalpine canopy plant *Paris japonica* Franchet (Pellicer *et al*., 2010), at 150 000 Mbp. Model plant species typically have smaller genomes, such as *Arabidopsis thaliana* 135 Mbp (Arabidopsis Genome Initiative, 2000) and rice (*Oryza sativa* L.) 500 Mbp (International Rice Genome Sequencing Project, 2005). Some of the large variation in genome size is due to polyploidy, which is extremely common, such that all plant genomes can be considered as deriving from one or more whole‐genome duplication events (Wendel, 2000), and the genome of *P. japonica* has been reported to be octoploid, accounting in part for its large genome size (Pellicer *et al*., 2010). However, major variations in genome size are also observed between closely related species of the same ploidy, such as *Vicia* (Bennett and Leitch, 2005; Chooi, 1971), probably due to differences in the prevalence of repetitive DNA sequence families.

Since the delivery of the Illumina genome analyser platform in 2006, the ability to generate sequence data has experienced more than a 1000‐fold increase with a corresponding average annual threefold reduction in the price of data generation (NIH: http://www.genome.gov/sequencingcosts/). This trend is expected to continue and has generated major opportunities for genotyping applications, specifically for highly multiplexed SNP‐based systems for implementation in GS and genomewide association studies (GWAS).

Target enrichment (based on sequence capture) and genome complexity reduction are two distinct approaches to GBS applications. A third potential option is the generation of a skim genome sequence with genotype imputation, and as the cost of sequencing continues to decrease, an increasing number of species will be amenable to whole‐ or partial genome sequencing in a cost‐effective manner. However, to date this approach has only been applied to model species with small, well‐characterized genomes such as *A. thaliana* and rice (Huang *et al*., 2010; Rowan *et al*., 2015).

Target capture is based on selective isolation of regions of interest prior to sequencing, with three main methods currently in common use: PCR amplification, molecular inversion probes (MIPs) and hybrid capture. PCR amplification uses primers to amplify regions of interest from the genome. The MIPs system (Hardenbol *et al*., 2003) uses primers that bind to sequences flanking a specific locus, forming a circular complex. Hybrid capture methods, either solid–liquid or liquid phase, isolate regions of interest from pre‐prepared DNA libraries. All current SNP chips are based on solid–liquid capture array systems, although liquid‐phase capture is gaining popularity (Gnirke *et al*., 2009; Mertes *et al*., 2011; Rohland and Reich, 2012).

Although target capture methods offer the advantages of specificity, reproducibility and enrichment of regions of interest, they do not detect novel variation, while requiring prior knowledge of genome structure and failing to adequately distinguish between similar genomic regions such as homoeologous and paralogous sequences (Winfield *et al*., 2012). Conversely, highly polymorphic regions may inhibit effective capture due to flanking polymorphisms and, invariably, target capture methods are costly due to a need for specialized design and manufacture.

Complexity reduction methods have been based on restriction enzyme digestion or transcriptome sequencing. One of the most popular and widely used methods is the GBS‐RAD (restriction site‐associated DNA) method of Elshire *et al*. (2011), which uses a restriction endonuclease to digest DNA samples before library preparation. Variations of the GBS‐RAD method have been extensively reviewed by Davey *et al*. (2011). Notably, this method suffers from large amounts of missing data (reviewed by Hirsch *et al*., 2014) and, due to generation of a significant number of dominant markers, is unsuitable for outbreeding species as it is unable to unambiguously identify heterozygous sites.

Sequencing of cDNAs (GBS‐t) has been described for genotyping applications in plants in a handful of studies in canola (Bancroft *et al*., 2011; Harper *et al*., 2012; Trick *et al*., 2009), wheat (Miller *et al*., 2016) and European ash (Harper *et al*., 2016) but has also been applied to the biomedical life sciences (Duitama *et al*., 2012; Quinn *et al*., 2013). Although variant splicing events can produce computational challenges (Duitama *et al*., 2012), which can be mitigated by alignment to a reference set of coding sequences (CDS reference genome), this system is also suitable for *de novo* reference assembly (Mizrachi *et al*., 2010). Furthermore, RNA shearing is less laborious and cheaper than most techniques for DNA fragmentation, and all detected sequence variants are genic. The major benefit of GBS‐t is to address the issue of varying genome size through normalization of the exome of different plant species. A comparison of *A. thaliana*, sorghum (*Sorghum bicolor* L.) and *Brachypodium distachyon* L. identified a conserved set of sequences present in mRNA of 60‐90 Mbp in size (Vogel *et al*., 2010). Furthermore, recent studies have revealed a trend for the majority of protein‐encoding genes of multiple plant species to be expressed in the majority of tissues and cell types (Sudheesh *et al*., 2016), supporting the broad applicability of GBS‐t approaches.

This study describes a GBS method based on transcript sampling that is cost‐effective and applicable to a broad range of crop species with differing levels of ploidy and degree of inbreeding/outbreeding, including perennial ryegrass, a diploid (2*n* = 2*x* = 14) outbreeding forage grass; phalaris, a putative segmental allotetraploid (2*n* = 4*x* = 28) outbreeding forage grass; lentil, a diploid (2*n* = 2*x* = 14) inbreeding grain legume; and canola, an allotetraploid (2*n* = 4*x* = 38) partially outbreeding oilseed. The diversity of species under investigation has demonstrated the versatility and effectiveness of the method.

## Results

**Figure 1:**
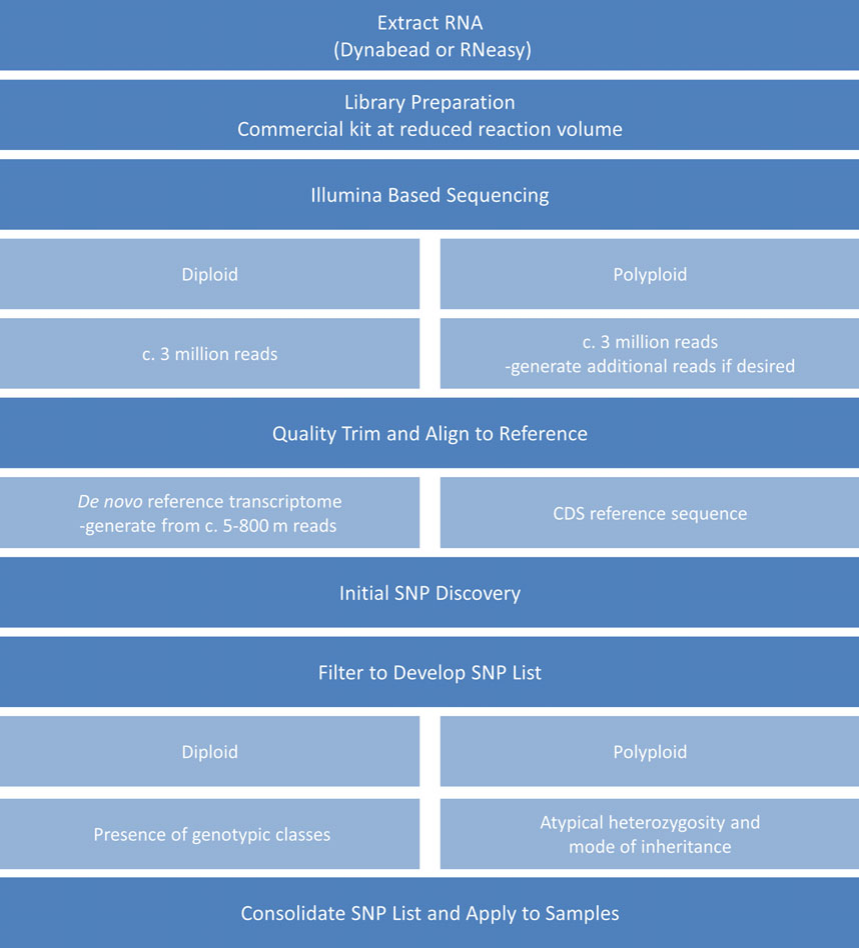
Overview of the GBS‐t workflow.

For all species, sampled transcripts were aligned to reference transcriptomes derived from previous RNA‐Seq assemblies or CDS files derived from full genome sequences. Both inbreeding and outbreeding crops at the diploid and tetraploid levels were selected to validate and exemplify the approach. An overview of the GBS‐t process is demonstrated in Figure 1.

### SNP identification in diploid species

A total of 182 lentil samples representing 38 ancestral genotypes were sequenced. A total of 973 648 994 high‐quality reads were generated, of which >90% aligned to the reference transcriptome assembly obtained from the cultivar Cassab. A total of 204 520 loci displayed both of the homozygous classes found in the consensus calls, and another 15 640 had only one homozygous class and at least four were heterozygous. A total of 11 817 loci had the opposing homozygous calls found within the line. As a result of these filters, a total of 231 977 SNPs were detected corresponding to 30 573 contigs. Of the total 30 573 contigs, 25 897 (85%) could be assigned to the seven chromosomes of lentil in a uniform manner.

For perennial ryegrass, variant site identification was performed as a two‐step process. A total of 83 samples were initially processed, representing a broad range of diversity. Indels, sites with >2 alleles called and sites with >50% missing data were removed, resulting in 1 079 770 variant sites. A requirement for presence of all three genotypic classes at a given locus reduced this number to 449 713. The second step involved SNP validation by genotyping of 95 full‐sibs from a two‐way pseudo‐testcross F_1_ mapping population. The SNP loci were evaluated for strict adherence to either 1:1 or 1:2:1 segregation ratios based on chi squared statistics, obtaining 50 216 loci. SNP loci that failed to segregate in the mapping population, but identified in all samples reliably with a homozygous genotypic class were also included, adding a further 89 556 loci to the prior set. In total, 139 772 SNP loci from a total of 11 787 reference transcriptome contigs and scaffolds were identified as a high‐confidence set.

### SNP identification in polyploid species

A collection of 285 phalaris samples that represented key parental genotypes from a breeding programme were assembled and processed. Using SAMtools mpileup, a collection of 6 008 286 variant bases were identified. However, SAMtools can only call genotypes for disomic SNPs. A range of other computational variant calling programmes were evaluated (Freebayes and GATK), but proved unsuitable for analysis of the putative segmental allotetraploid. Manual inspection of individual variant base positions and associated haplotypic configurations were therefore used to identify loci with either five genotypic classes (conforming to tetrasomic inheritance, from chromosomes with an autopolyploid constitution) or three genotypic classes (conforming to disomic inheritance, from chromosomes with an allopolyploid constitution).

All of the currently available variant calling software packages are designed for genomes of consistent ploidy and currently do not provide genotype identification from data sets with mixed inheritance patterns as described above. A simple computational process based on a custom R script was developed for variants identified using SAMtools mpileup, to return the bases aligned at the specific designated base location and call allelic proportions based on the aligned observed bases. Genotypes were called based on a ±5% variation around the expected frequencies. For the alternative allele, <5%, 20–30%, 45–55%, 70–80% and >95% were the designated bins relating to the presence of 0, 1, 2, 3, 4 copies of the given allele. From the 6 008 286 initially identified variant bases, a total of 89 738 sites from the 285 samples could be reliably called using these parameters. Further analysis of the segregation ratios for these SNPs revealed a 4:1 ratio of tetrasomic to disomic inheritance patterns.

A total of 575 lines of canola, representing both spring‐type lines primarily from Australia and winter‐type lines from various geographic locations, were subjected to GBS‐t. Initial filtering of the entire data set resulted in 104 809 high‐confidence SNPs. Examination of population structure revealed significant differences between spring‐ and winter‐type lines, and so filtering was performed separately, resulting in 123 469 SNPs in the spring‐type samples and 179 656 SNPs in the winter‐type samples. Of these, 76 270 were common between both groups, resulting in a total of 226 855 unique SNPs.

**Figure 2:**
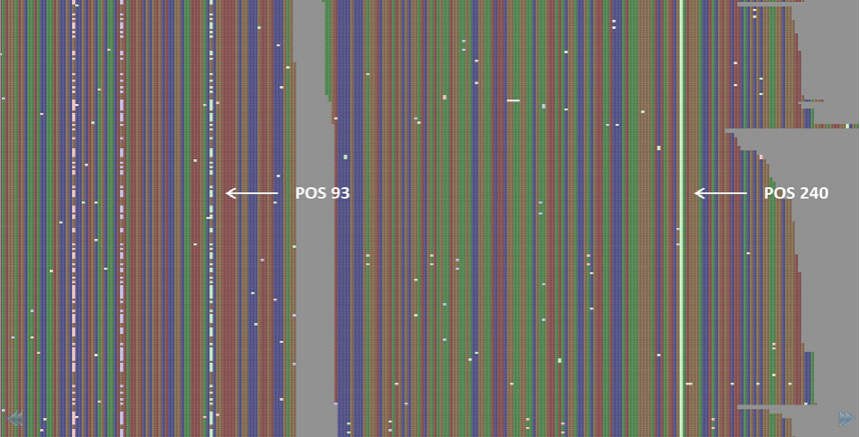
View of read alignments using Tablet for the gene BnaA08g04820D in the sample AG‐Comet BAM file. The SNP called at position 93 is putatively misaligned due to local similarity between the A and C subgenomes of canola, while the SNP at position 240 is putatively homozygous for an alternate base.

Reprocessing all bam files with mpileup using this SNP list to provide complete genotype profiles and filtering across the whole data set resulted in 185 787 high‐confidence SNPs from 521 individuals using stringent filtering parameters, including an increased minor allele frequency (MAF) of 0.05 to account for reduced sample size due to separate processing of spring and winter types, removal of individuals with large amounts missing data, and omission of loci with a high degree of heterozygosity. SNP loci with a >0.4 proportion of heterozygous calls were deemed to be the result of misalignment between homoeologous sequences on the A and C subgenomes, and so were subsequently removed. This was performed on a locus‐by‐locus rather than gene‐by‐gene basis, as evidence was found of well‐aligning SNPs within the same gene. As an example, in Figure 2, the putative homoeologous sequence variant at position 93 is repeated across the entire data set (99% of all samples), but the predicted SNP at position 240 was genotyped as heterozygous in only 44.7% of samples. Read depth did not have an impact on genotypic calls, as the average values for the two positions were 295 and 230, respectively.

### Analysis of data completeness and reproducibility

**Figure 3:**
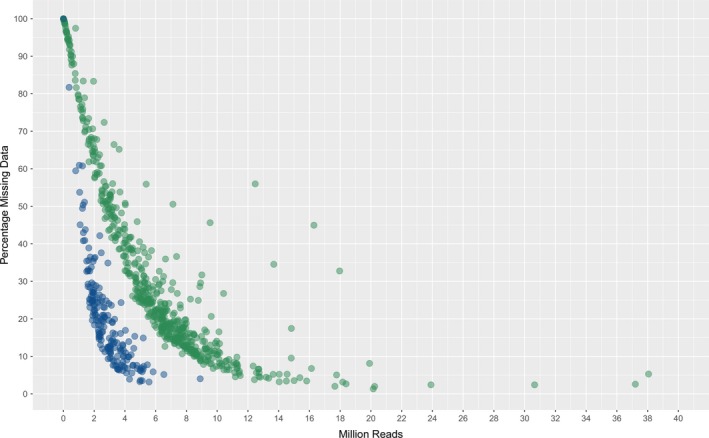
Percentage missing data and number of reads generated per sample for perennial ryegrass (blue) and canola (green).

An evaluation of missing data compared to number of sequence reads generated was performed for representative samples from the diploid (perennial ryegrass) and polyploid (canola) species. The diploid species generated a profile where c. 3 million sequencing reads (c. 1% of a HiSeq run‐lane) resulted in missing data across the 139 772 SNP markers of c. 15%, while for the polyploid species a much higher level of missing data (c. 50%) was observed (Figure 3). An increase to c. 7 million sequence reads was required to achieve c. 15% missing data. For both species, complete data were not obtained from any sample regardless of number of sequence reads.

**Figure 4:**
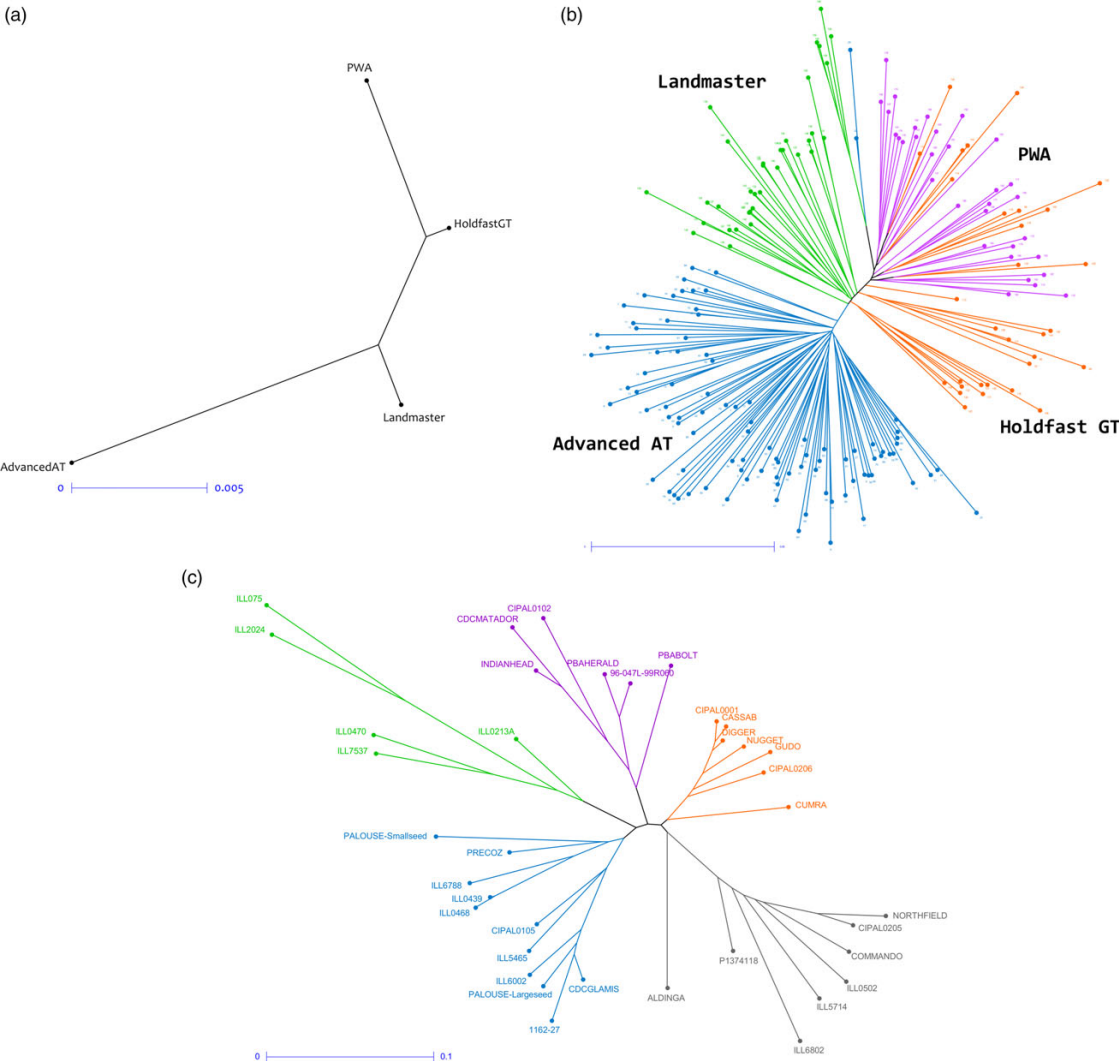
NJ dendrograms for phalaris and lentil calculated using Nei's pairwise genetic distance. For phalaris, relationships between populations (a) and individuals (b) are displayed. The dendrogram showing individuals displays samples from the Advanced AT cultivar in blue, Landmaster in green, Holdfast GT in orange and PWA in purple. Relationships between lentil parental cultivars are also displayed, based on calculations using the same method (c).

Reproducibility of the GBS‐t approach and its suitability for genetic diversity assessment was evaluated using the phalaris and lentil data sets. Multiple samples were available from four phalaris varieties (95 Advanced AT, 31 Holdfast GT, 35 Landmaster, 32 phalaris winter active [PWA] to a total of 193). Nei's genetic distance was calculated for the complete data set, and neighbour joining (NJ) dendrograms were generated (Figure 4a,b). All samples that originate from a given cultivar were clustered together (Figure 4b), with the exception of 13 Holdfast GT samples located within the PWA group. However, Holdfast GT has common parentage with the PWA material, so a degree of genetic overlap is expected.

Genetic distance between lentil genotypes was quantified (Table S1), and five clusters of significant size were obtained (Figure 4c). Based on the calculation of genetic distance between 38 lines, the most divergent pair were Northfield and ILL075 (Nei's coefficient value 0.391; Table S1) while ILL0468 and ILL0439 exhibited minimum genetic distance (Nei's coefficient value 0.012, Table S1). A weighted NJ tree was generated (Figure 4c) and average level of heterogeneity within each line was calculated to be 4.35%, which is expected as most of these lines were at the F_7_/F_8_ generation level following single seed descent.

### Genomic coverage of markers

Analysis of the distribution of identified SNPs across the genome was performed for two species. Of the four species, only canola provided a full reference genome sequence, to which sampled SNP loci were mapped. For a second evaluation in perennial ryegrass, SNPs identified as segregating in the pair cross‐derived full‐sib population were assigned to genetic map locations, as a proxy exercise for genomic location.

**Figure 5:**
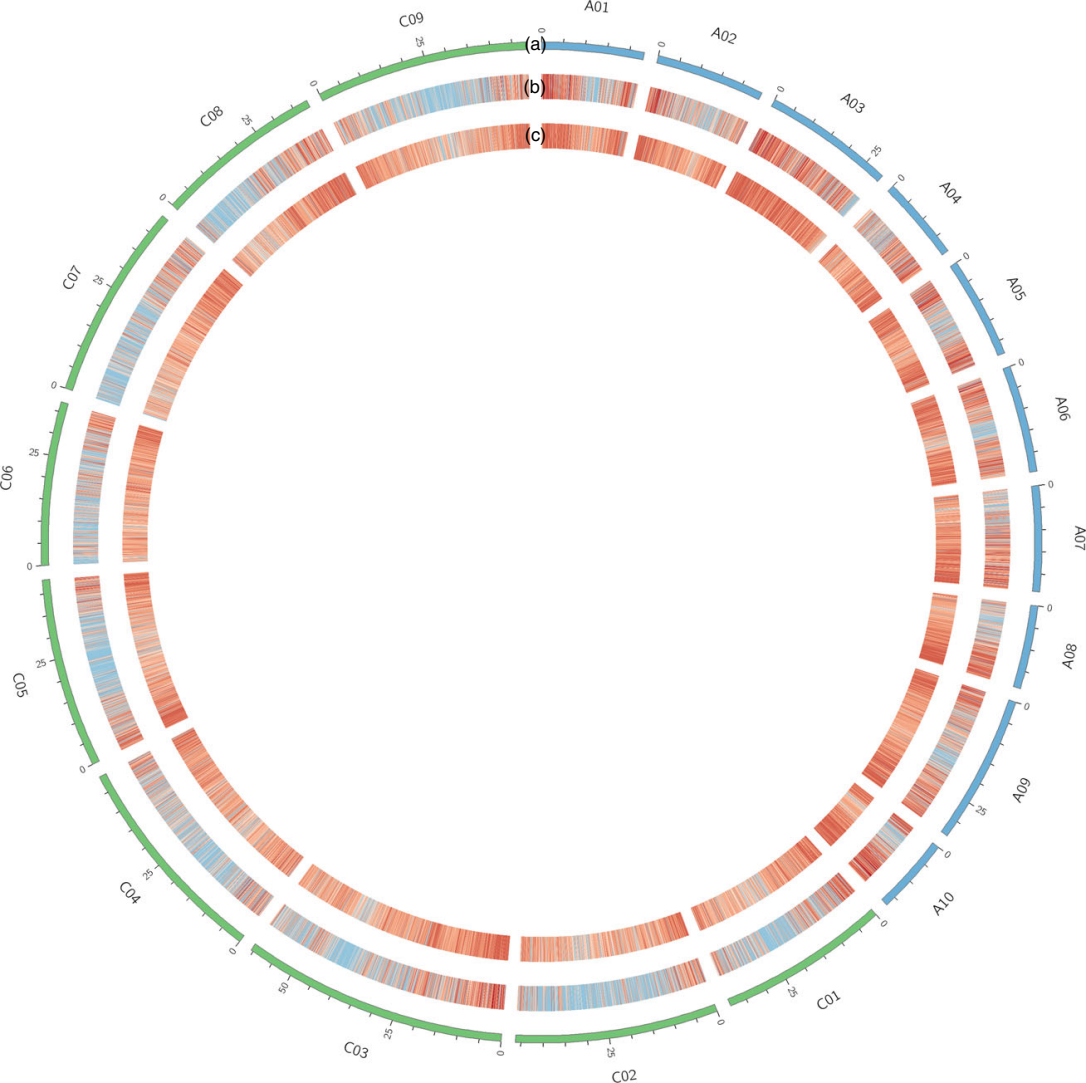
Canola SNP and gene density heatmaps. Red indicates high density, and blue indicates low density. Tracks displayed are as follows: (a) the canola karyotype; (b) density of SNPs discovered through GBS‐t; and (c) gene density based on the CDS reference genome.

**Figure 6:**
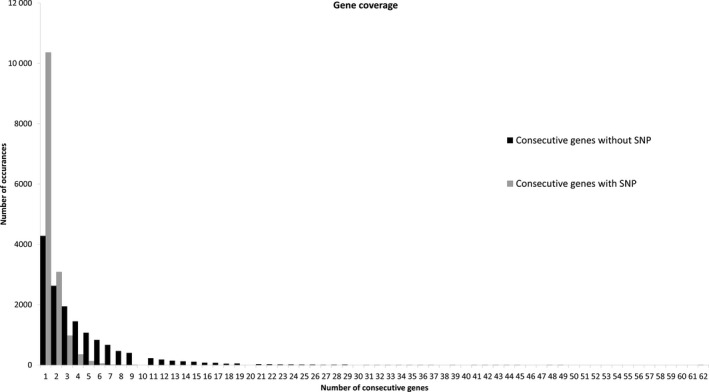
Frequency histogram of distribution of canola genes containing assayed SNPs. Black bars indicate the frequency of consecutive genes without SNPs. Grey bars indicate the frequency of consecutive genes with at least one SNP.

Single nucleotide polymorphism density and gene density were compared for canola (Figure 5) and are closely correlated, with higher and lower gene density near to the telomeres and predicted centromeres, as expected. A frequency histogram of consecutive genes containing SNPs was also constructed to visualize SNP distribution across the genome (Figure 6). Just under a quarter of genes (22 039/88 428) with a known location in the reference genome were included. The frequency of consecutive genes without a SNP decreased rapidly, with 90% (19 835) of SNP‐containing genes located within 17 genes or less of one another (Figure 6). The largest observed gap was 62 genes, occurring once on chromosome C01.

**Figure 7:**
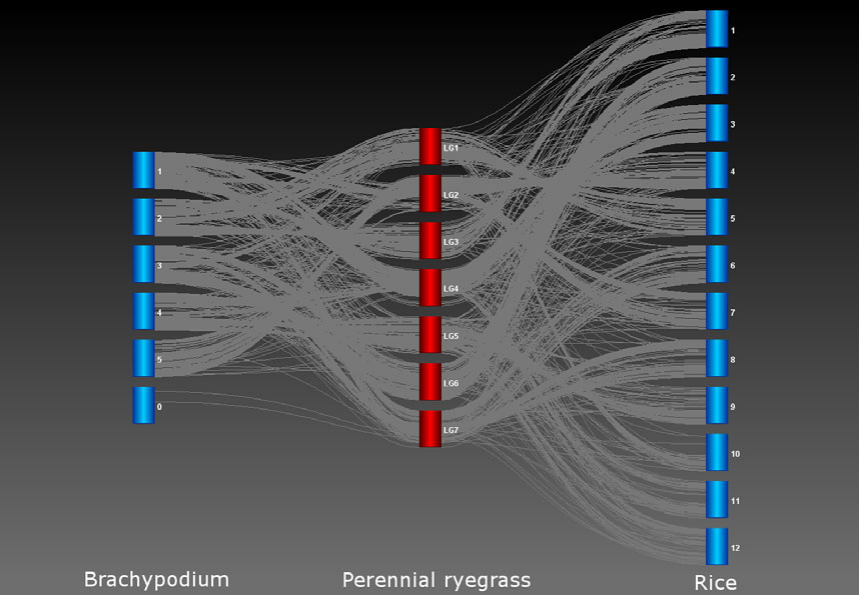
Comparative alignment of loci on the perennial ryegrass genetic map with genome locations of matching DNA sequences in the genomes of rice and *Brachypodium distachyon*.

For perennial ryegrass genetic map construction, a single SNP per segregating reference contig was selected for map construction. After the two sets of parental linkage groups for the two‐way pseudo‐testcross structure were generated, specific bridging markers were added to generate a consensus order. This resulted in 2018 genic SNP loci being ordered at high density on seven linkage groups (Figure 7 and Table S2). In addition, the corresponding gene sequences were compared to the reference genomes of rice and *B. distachyon* and a comparative map alignment was generated (Figure 7).

## Discussion

The variety of genome sizes, SNP frequencies, levels of ploidy and reproductive habit that are characteristic of crop plants provide substantial challenges for the development of highly multiplexed marker tools. Effective implementation of GS in a variety of crops requires a variable number of markers and a range of cost structures will be justifiable, so it is unlikely that a single marker technology will ever be applicable to all species and breeding systems. However, the method described here provides a high‐throughput, cost‐effective solution that could be applied to many crop species. The key benefit of GBS‐t is the ability to reduce genome complexity to the level of a partial exome irrespective of nuclear DNA content, prevalence of repetitive DNA sequences and ploidy level, while maintaining sufficient resolution and conservation of the gene sequences, greatly assisting with sequence assembly and SNP identification. In addition, GBS‐t is suitable for reference assembly in genomic ‘orphan’ species. Approximately 500–800 million sequence reads from a range of tissues are sufficient to generate a reference which can be relatively easily assembled, as shown by Baillie *et al*. (2017), Shinozuka *et al*. (2017) and Sudheesh *et al*. (2016).

### Advantages of GBS‐transcriptomics over related systems

The GBS‐t approach has substantial advantages over other GBS methods. Unlike target capture methods, costly probe design is not required and the system can be developed and implemented with a relatively small investment. Costs will decrease further due to the continued trend of reductions in the price of sequencing, which is also true of other GBS systems but not SNP chip systems. Reduction of reagent volumes used in library preparation has lowered the cost of the protocol, and further reductions in price could be realized through more custom procedures of sample preparation such as use of automation, further increasing the high‐throughput capacity of this method.

Broad‐scale adoption of target capture methods has been further inhibited by a need for prior knowledge of the genome of interest, while the commonly used GBS‐RAD systems are prone to problems due to variable genome size and the effects of high‐sequence polymorphism on restriction site access. This has led to large amounts of missing data, over‐representation of homozygous genotypes and an excess of dominant markers, which is suboptimal for outbreeding species. Even inbreeding plants, where the tool was developed, may show significant levels of residual heterozygosity, as shown in the present study by canola and lentil, leading to compromised genotypic profiles. Additionally, breeding methods such as GS and optimal haploid value selection (Daetwyler *et al*., 2015) can make accurate selections in inbreeding species still in a heterozygous state. Consequently, the ability to accurately call heterozygous genotypes enables earlier breeding and selection decisions, decreasing generation intervals, and increasing genetic gain.

While previous studies applying transcriptome data for SNP genotyping have typically generated c. 30 million reads per sample (Bancroft *et al*., 2011; Miller *et al*., 2016), higher multiplex ratios can be exploited as has been demonstrated here, indicating that 3 million reads is more than sufficient to obtain largely complete information for diploid species, while for more complex genomes such as allotetraploid canola, additional sequence depth is desirable but not essential. In wheat, an allohexaploid species, c. 50% missing data are still sufficient for accurate genotype imputation (Rutkoski *et al*., 2013). The data from the present study probably indicate that the canola genome, possibly due to its polyploid nature, may have a higher level of hemizygosity or more variable gene expression. A total of two samples from the canola data set had in excess of 36 million reads but still had c. 3 and 5% missing data. Variations in gene number as well as structural variants will impede all SNP genotyping systems and requires a pan‐genome reference to overcome these alignment issues.

Reproducibility of the method has been demonstrated by the robust relationships revealed from genetic diversity analyses. In the case of phalaris and lentil, many of the close affinities observed in the NJ tree reflect the known breeding history (where available) of the respective lines. In lentil, for example, PBA‐Bolt and PBA‐Herald were both derived from 96‐047L‐99R060, consistent with their close affinity. Interestingly, CIPAL0102, which was derived from both Indianhead and Northfield (located in a separate cluster), is located closer to the former, indicating a higher proportion of Indianhead‐derived genes.

Genomic analyses of marker coverage revealed even sampling of genes throughout the genome. In canola, the distribution of high‐confidence SNPs matched gene density, such that just over a quarter of genes with even distribution were represented, with few large gaps. The most common scenario was a single gene without SNPs flanked by two genes possessing on average eight SNPs, thus generating enough information for marker–trait associations in the majority of cases. Blackleg disease resistance is of great interest to canola breeders due to the severity of the disease. This study found that while three putative blackleg resistance genes (Fomeju *et al*., 2015; Larkan *et al*., 2014, 2015) did not contain SNPs, numerous surrounding genes did (Figure S1a,b), which should provide sufficient resolution for GS. In the absence of a full genome sequence, genetic linkage mapping was used to obtain similar verification for perennial ryegrass, leading to establishment of a high‐density genetic map with relatively even coverage. Comparative genomics with model Poaceae species confirmed these observations at the level of conserved macro‐colinearity. GBS‐t based only on leaf tissue has therefore been shown to provide sufficient coverage across the genome. Although the use of a single tissue type for transcript sampling will exclude some genes due to tissue‐specific expression, this method resulted in even sampling across gene classes in the canola genome (Figure S2) and a more comprehensive regime of sampling is impractical and costly. Microarray‐based studies of *A. thaliana* (Ma *et al*., 2005) and soya bean (Le *et al*., 2012) estimated c. 70% of genes to be expressed in leaf tissue, but due to sensitivity limitations, this value is probably underestimated. RNA‐Seq‐based studies have obtained higher estimates, including for species involved in the present study, lentil (97%: Sudheesh *et al*., 2016) and canola (96%: Chalhoub *et al*., 2014). Concerns over tissue‐dependent gene expression may be one of the reasons transcriptome‐based GBS has not been broadly adopted by the plant genomics community, but the present study has demonstrated in four diverse species that this is unlikely to compromise the application of GBS‐t for studies dependent on marker–trait correlation such as GWAS and GS.

Genomic selection offers major advantages for breeding, particularly for species with long generation times. Tree breeding rapidly adopted GS, with a biparental population of domesticated apple achieving a prediction accuracy of 0.7 for flowering time using 2500 SNPs (Kumar *et al*., 2012b), while *Eucalyptus* achieved a prediction accuracy of 0.55 for pulp yield using 3300 DArT markers (Resende *et al*., 2012a), and pine observed a prediction accuracy between 0.65 and 0.75 for diameter breast height and total height using 4825 SNP markers (Resende *et al*., 2012b). GBS‐t has also proven capable of providing sufficient markers for applications such as GS and GWASs, which in canola typically use c. 25 000 markers (Hatzig *et al*., 2015; Jan *et al*., 2016) or less (13 973 SNPs Fomeju *et al*., 2015). The GBS‐t method resulted in 185 787 high‐confidence SNPs in the canola sample set and c. 100 000 or more in all other species, providing a higher degree of resolution than typically used for GS or GWAS, at similar cost as other genotyping methods including GBS‐RAD and SNP chips.

### Potential limitations of GBS‐transcriptomics

A potential limitation of the method is the influence of allele‐specific gene expression (ASE). Expression imbalances may be exclusive, such that only one allele is expressed, or preferential, such that both alleles are expressed, but at ratios deviating from 1:1. There have been variable estimates of ASE in plants, with 43–53% (Springer and Stupar, 2007) and 60% (Guo *et al*., 2008) of genes found to exhibit some degree of ASE in maize, with 9% reported to be exclusive (Altrogge *et al*., 2016). However, a global analysis of ASE in *A. thaliana* detected 13% of genes with significant effects (Zhang and Borevitz, 2009), and in rice, the equivalent value was 27.5%, of which 3.7% were exclusive (Song *et al*., 2013). For GBS‐t, exclusive allelic expression will prevent access to some loci, but fortunately, only a minority are likely to be affected and a reasonable depth of sequence read sampling should be sufficient to recover both alleles from genes with preferential expression. For extreme expression ratios of 90:10, the probability of sampling at least one copy of each allele is 0.65 for 10 reads, and 0.88 for 20 reads (Table S3). While it is not possible to completely eliminate the effect of ASE on SNP genotyping, it can be mitigated by increasing sequencing depth or applying more stringent filtering which will lead to fewer but higher confidence SNPs. Either of these measures can be applied if desired but are not essential as sufficient loci should still be available for GWAS and GS, and needs to be evaluated as a trade‐off against cost.

A second potential limitation of GBS‐t is the inability to capture sequence variation in noncoding regions including regulatory regions and introns. In cases where genotyping of specific regions is necessary, such as for marker‐assisted selection, GBS‐t may not be suitable. Required marker density for accurate implementation of GWAS and GS depends on a number of factors including effective population size, trait complexity, heritability as well as LD. Inbreeding species such as lentil and canola are expected to display extensive LD, while outbreeding species close in structure in ecotypic populations (such as perennial ryegrass and phalaris) have shown a more rapid decay of LD (Ponting *et al*., 2007). However, modern synthetic populations of perennial ryegrass based on limited numbers of parental genotypes exhibit reduced decay of LD (Auzanneau *et al*., 2007). While increased LD means fewer markers are required, increased marker density generally increases prediction accuracy, particularly for complex traits with low heritability (Duangjit *et al*., 2016), although this consideration should be balanced against an increased computational burden.

Single nucleotide polymorphism identification in polyploid plant species has been highlighted as an issue in this study due to an absence of appropriate software. As many crop species are polyploids, and whole‐genome duplications have been common in the evolutionary histories of plants (Wendel, 2000), this is a major challenge in crop improvement. The choice of software will also influence the outcomes of the SNP calling process: a comparison of SAMtools, Freebayes and GATK revealed that only 1.4% of variant bases were identified as common between the three programmes (Clevenger *et al*., 2015). Clevenger *et al*. (2015) also identified SAMtools as the most aggressive variant caller, consistently calling the least false‐negative and false‐positive homoeologous SNPs as well as identifying most of the rare variants. However, calling genotypes in a putative segmental allopolyploid genome, such as that of phalaris, is still a nontrivial undertaking as both disomic and tetrasomic patterns of inheritance need to be addressed. The segregation ratios identified from the phalaris data in the present study indicate the presence of both auto‐ and allotetraploid chromosomal configurations, consistent with some previous studies of chromosome pairing in this species (Putievsky *et al*., 1980). Although segmental allopolyploidy provides the simplest explanation for the observed patterns, it is also possible that some homoeologous sequences in a full allopolyploid constitution have been assembled into a single structure within the reference transcriptome, while others have remained separate. There is also the possibility that significant homoeologous recombination occurred during phalaris evolution, resulting in SNPs common to both subgenomes that appear to segregate in an autopolyploid manner. Further studies are required to comprehensively characterize the structure of the phalaris genome.

Single nucleotide polymorphism identification is further complicated in polyploids due to multiple gene copies, facilitating functionalization and resulting in false‐positive SNPs caused by misalignment between homoeologous regions. Issues of misalignment can be reduced by filtering for high mapping quality and unambiguous alignment; however, imperfect reference genomes will prevent the detection of all such regions due to sequence collapse. Another approach is to filter for unexpected levels of heterozygosity. Although some residual heterozygosity has been observed, canola is an allotetraploid with disomic inheritance, and the majority of cultivated varieties are highly inbred or derived as double haploids with one‐step full homozygosity. For this reason, relatively low levels of heterozygosity are expected, and observation of unusually high levels of heterozygosity, as detected by GBS‐t, could indicate the effects of misalignment. Evaluation of such effects should be undertaken on a locus‐by‐locus basis rather than a gene‐by‐gene basis, as local similarity between the homoeologous sequences will vary within the gene. Along with the expansion of SNP numbers achieved by calling SNPs separately in spring and winter types of canola, SNP calling from data sets such as these needs to be an iterative process in which the data generated are screened for population structure and then reprocessed. More broadly, SNP identification in canola exemplifies issues inherent in not only polyploids, but also multicopy gene families. As canola has undergone whole‐genome duplication events within each diploid subgenome, every gene will have several highly similar sequences and can be considered a gene family. Approaches described here for canola will therefore have relevance across most plant species.

## Conclusions

Whilst GBS‐transcriptomics has been described before, this study has exemplified the broad applicability of GBS‐t to crop species with varying genome sizes, ploidy and breeding habit. It has shown that GBS‐t can be a cost‐effective method by limiting sequencing reads to c. 3 million per sample and reducing library preparation reaction volumes, as well as using high‐throughput preparation techniques including tissue grinding and library preparation in plate format. Not only is the cost comparable to other GBS‐t methods, but it is without limitations in its freedom to operate and delivers c. 100 000 or more evenly spread SNP markers from single leaf tissue extraction. Importantly, the bioinformatics process should be iterative and consider the structure of the genome in question to fully utilize the generated sequence data. GBS‐t is particularly well suited to plant species with large genomes, those lacking significant genomic resources or whole‐genome sequences, and with research and breeding communities of modest size. Although species with small, fully defined genomes and predefined SNP catalogues may be amenable to skim whole‐genome sequencing as technology advances and costs fall, the GBS‐t technique is likely to still be of value in the interim.

## Experimental procedures

### Plant material

Plant materials were selected for all species to encompass a broad range of genetic diversity (Table S4).

RNA extraction for all perennial ryegrass, phalaris and canola samples was performed using a modified method from Kumar *et al*. (2012a). The protocol was automated with tissue disruption performed in a 96 well‐plate format (using a QIAGEN [Hilden, Germany] mixer mill or Spex [Metuchen, NJ] SamplePrep genogrinder) and then mRNA extraction performed by Dynabead extraction (Life Technologies, Carlsbad, CA) using a Biomek FXp (Beckman Coulter, Brea, CA) robotic platform. Lentil RNA extraction was performed using the RNeasy (QIAGEN) protocol following manufacturer's instructions.

Following mRNA extraction, sequencing library preparation was in all instances performed using the SureSelect stranded RNA library preparation kit (Agilent Technologies, Santa Clara, CA) following manufacturer's instructions, with a reduction in reaction volume. Libraries were evaluated using the TapeStation 2200 platform (Agilent Technologies), pooled and quantified using the KAPA library quantification kit (KAPA Biosystems, Wilmington, MA), with the exception of canola, which was quantified using the Qubit (Life Technologies) Fluorometer according to manufacturer's instructions. Sequencing data were generated using Illumina (San Diego, CA) HiSeq 2000 or 3000 or NextSeq 500 systems.

### Bioinformatic data analysis

In all instances, visualization of alignments was performed using Tablet (Milne *et al*., 2013). For lentil, perennial ryegrass and phalaris, reference transcriptomes have already been described (Baillie *et al*., 2017; Shinozuka *et al*., 2017; Sudheesh *et al*., 2016). To provide a common standard across all species, the CDS file from the canola reference genome sequence (Chalhoub *et al*., 2014) was used as a proxy for a reference transcriptome. A simple *in silico* SNP genotyping process was implemented, involving initial fastq sequence quality trimming using a custom perl script as well as cutadapt v1.4.1 (Martin, 2011). The trimmed sequence data were then aligned to the reference exome using BWA and the mem algorithm (Li, 2013) and then converted to a BAM file and ultimately a vcf file using SAMtools view, mpileup, bcftools, vcfutils (Li *et al*., 2009) and vcftools (Danecek *et al*., 2011). Following the initial discovery process, a defined SNP list was developed and provided in the mpileup SNP filtration in all instances. As a generic filtering pipeline using mpileup, bcftools, vcfutils and vcftools, variants were called while ignoring indels, filtering for a minimum read depth of 5, a minimum of four alternate bases before calling a SNP, accepting only bi‐allelic SNPs, and only accepting SNPs with a MAF of 2%, and maximum missing data of 50%.

As vcftools does not consider nor accept population information corresponding to samples to allow different filtering options across and within populations/varieties (e.g. to retain SNP loci if the MAF criterion is satisfied in at least one population, but not the whole data set), a custom in‐house script was developed for data filtering. In‐house bash scripts were developed to extract the bi‐allelic genotypes (as a matrix) from the generated vcf files with a corresponding matrix of read depths (for subsequent missing data analysis). In‐house R scripts were also developed and used for MAF estimation and missing genotype data filtering, taking into account SNP loci performance within or between populations. For genotypes of canola, BAM files were sorted into spring and winter types due to the presence of significant population structure, and variants were called separately. The previous bioinformatic pipeline was altered such that the MAF threshold was adjusted to 5% within each population to allow more stringent variant calling. The two resulting SNP lists were then consolidated and all BAM files rerun through SAMtools mpileup to produce a complete SNP profile, removing SNPs found to be tri‐allelic between spring and winter types.

### Additional filtering for diploid species

Up to five seedlings were genotyped for each lentil line, and a consensus was determined based on the most common genotype observed within the line. SNP loci for which both homozygous classes were observed across the consensus calls were retained as a high‐confidence set. SNP loci for which only one homozygous class was observed in the consensus calls, but at least four heterozygous consensus calls were also observed, were retained. Additionally, SNP loci for which both homozygous calls were not observed across the consensus calls, but both homozygous classes were found within one or more lines were also retained for future SNP calling.

### Additional filtering for tetraploid species

For both polyploid canola and phalaris, additional SNP filtering was performed after the initial SNP list was developed. SNP loci with >0.4 proportion of heterozygous calls were deemed to be the results of misalignment between highly similar regions of the subgenomes and were therefore removed.

Due to the putative segmental allopolyploid nature of the phalaris genome, allele frequencies were called rather than bi‐allelic genotypes and genotypes were scored based on binned allele frequencies. Allele frequencies not conforming to the defined bins were relabelled as missing data, and SNP loci were retained where three or more different pseudo genotype calls were present across samples. Allele frequencies at these SNP loci were used for all further analyses.

### Validation of the GBS‐transcriptomics method

Genetic map construction in perennial ryegrass was performed using Joinmap v4.1 with Haldane regression mapping and a LOD threshold of 8. Parent‐specific linkage maps were then merged using mergemap (http://www.mergemap.org) through the presence of bridging markers. Transcript sequences generated in the reference assembly were compared to reference assemblies from rice (*Oryza sativa* japonica group assembly build 4.0) and *B. distachyon* (version 1.0) using BLASTN (Altschul *et al*., 1997) and the match with the lowest E‐value was recorded. Comparative genome alignment between the model species and the merged ryegrass genetic map was performed using Strudel v1.12.03.20 (Bayer *et al*., 2011).

To assess levels of genetic diversity within lentil and phalaris, Nei's pairwise genetic distance was calculated, and subsequently, genetic diversity within lentil and phalaris was calculated using StAMPP (Pembleton *et al*., 2013). NJ dendrograms were generated based on the method proposed by Saitou and Nei (1987) and displayed in DARwin v6.0.5 (Perrier and Jacquemoud‐Collet, 2006).

Once a consolidated list of SNP loci had been generated, examination of SNP and gene density for canola was performed through heatmap construction using Circos (Krzywinski *et al*., 2009), excluding SNPs assigned to genes with an unknown or ‘random’ location in the reference genome.

## Conflict of interest

The authors declare no conflict of interest.

## Author's contributions

MMM, RB, MCD, PV and SS prepared plant materials, performed RNA extraction and sequencing library preparation. MMM, SK, SS, LWP, HS and NOIC performed the data analysis. MMM, NOIC, SK, JWF, HDD and GCS all conceptualized the project and assisted in drafting the manuscript. All authors read and approved the final manuscript.

## Supporting information


**Figure S1** Canola SNP coverage of the agronomically important trait blackleg disease resistance.

**Figure S2** Gene ontology analysis in canola.

**Table S1** Lentil genetic distance matrix.

**Table S2** Perennial ryegrass genetic map information and comparative genome analysis to rice and *B. distachyon*.

**Table S3** Allele‐specific expression (ASE) sampling probabilities.

**Table S4** Description of lines used in each of the 4 species.
