# Supplementary material for: Genotyping‐by‐sequencing through transcriptomics: implementation in a range of crop species with varying reproductive habits and ploidy levels
Source: Plant Biotechnol J. 2017 Oct 13;16(4):877–89. doi: 10.1111/pbi.12835 (PMC5866951; doi:10.1111/pbi.12835)
Supplement: Supplementary file 1 — Figure S1 Canola SNP coverage of the agronomically important trait blackleg disease resistance. [file PBI-16-877-s005.docx]

**Figure S1 – Canola genome browser blackleg resistance genes**


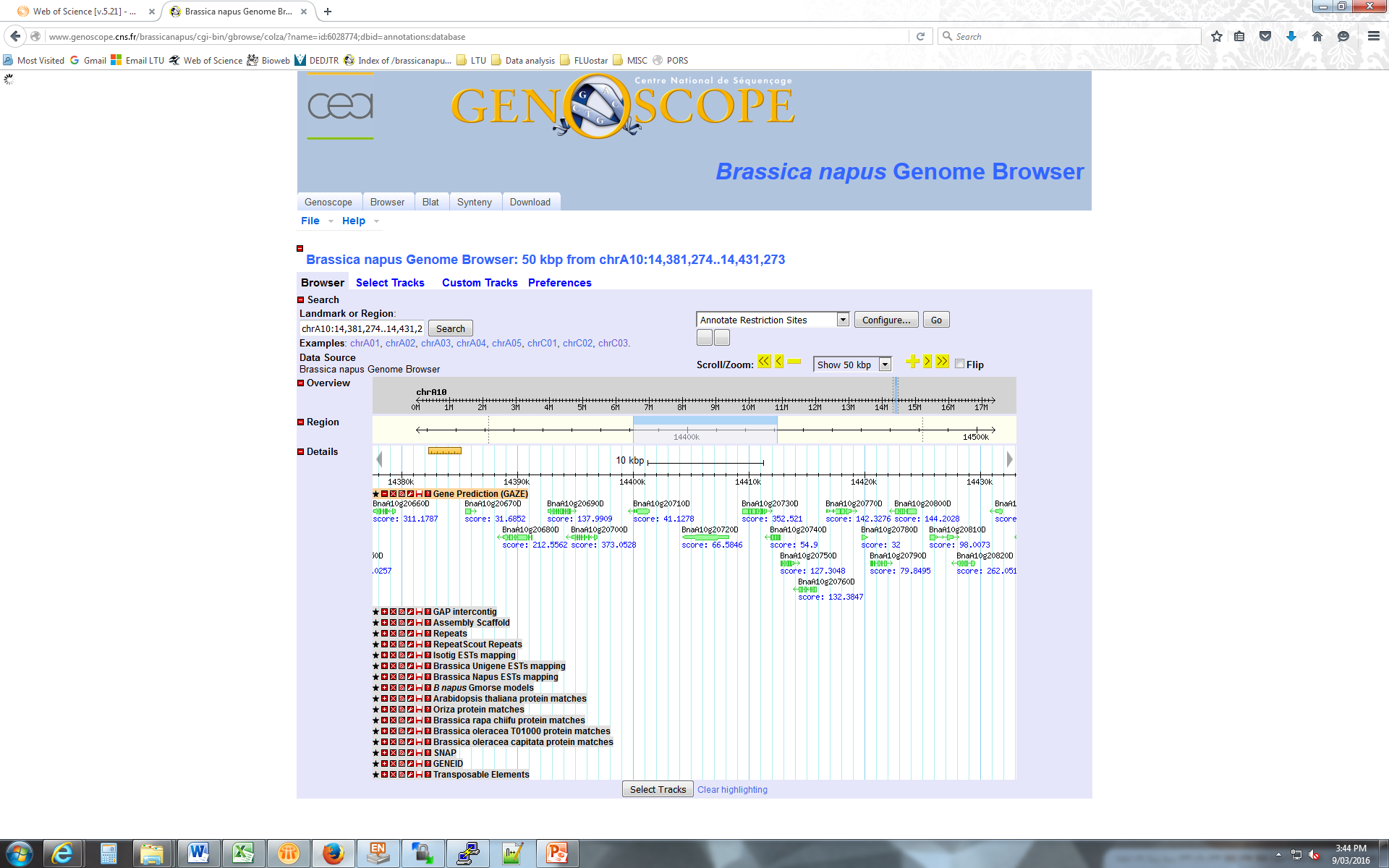


**Figure S1a** Candidate gene for the blackleg resistance gene Rlm2/LepR3 (BnaA10g20720D) is circled in red. Genes with SNP are underlined.


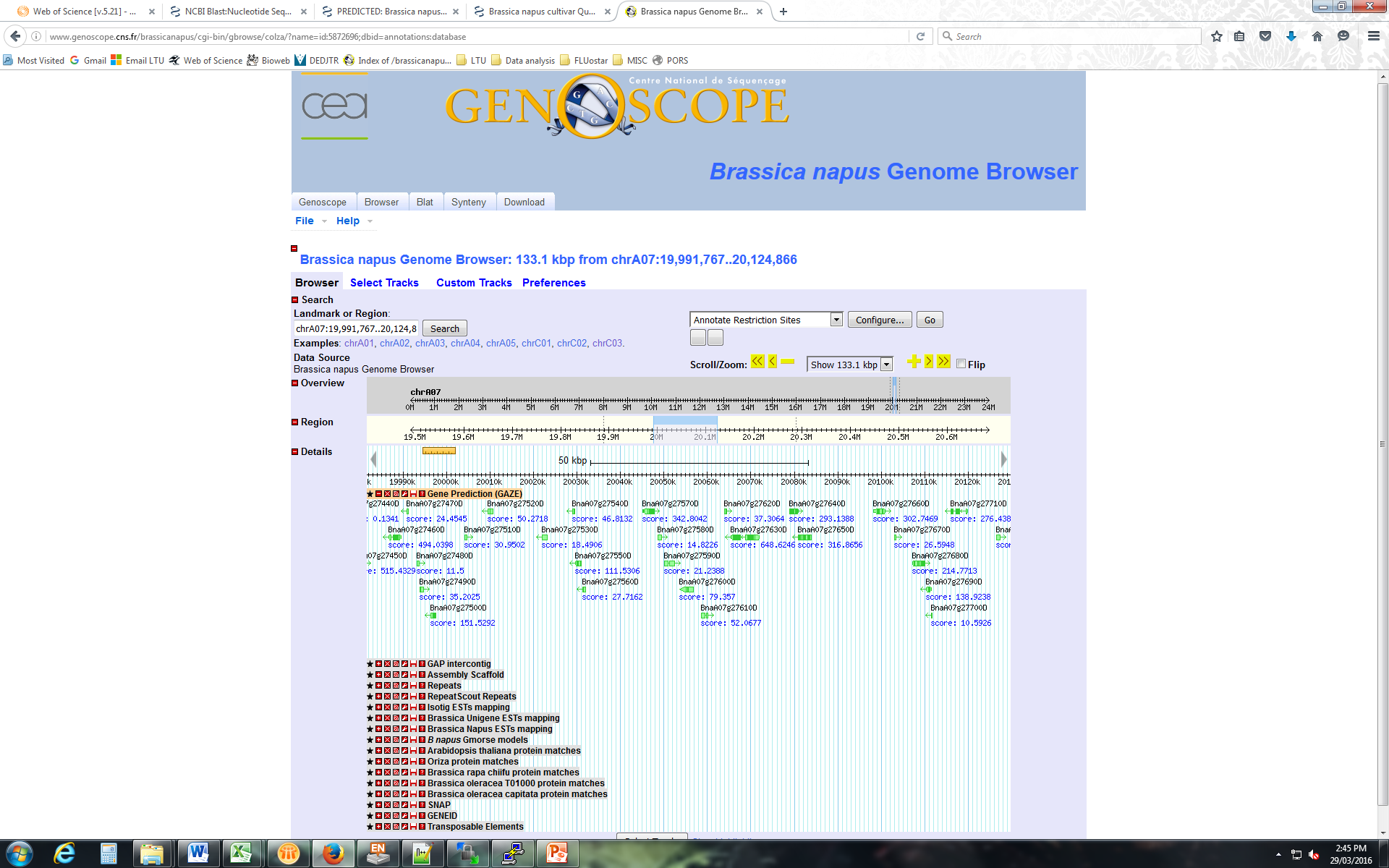


**Figure S1b** Two of several candidate genes for the blackleg resistance gene Rlm1 (BnaA07g27530D and BnaA07g27670D) and are circled in red are. Genes with SNPs are underlined.

Blackleg disease resistance genes are of great interest for canola breeding programs due to the severity of this disease. To examine the coverage of genes of interest using GBS-t, candidate genes for blackleg resistance were identified and the regions harbouring putative genes were examined for the presence of SNPs. Specific regions of interest in the canola genome were identified and examined using the Genoscope *Brassica napus* genome browser (http://www.genoscope.cns.fr/brassicanapus). BnaA10g20720D is a likely candidate for the blackleg resistance gene Rlm2/LepR3 located on chromosome A10 (Larkan et al 2014, 2015). Although no SNPs were discovered within the gene itself, four surrounding genes contained 41 SNPs, including 3 SNPs in BnaA10g20730D, the downstream gene (Figure S1a). BnaA07g27530D and BnaA07g27670D are two of several candidate genes for the blackleg resistance gene Rlm1 located on chromosome A07 (Fomeju et al 2015). The genes of interest did not yield SNPs but 5 surrounding genes with a combined 59 SNPs were identified (Figure S1b). This should provide sufficient marker information for selection related to this trait since empirical studies have demonstrated significant LD in canola (Wu et al 2016). It is possible that the nature of this gene is presence absence or highly hemizygous, such that it may not be possible to detect variation within the population based on SNPs within the gene itself.
