# Supplementary material for: Genotyping‐by‐sequencing through transcriptomics: implementation in a range of crop species with varying reproductive habits and ploidy levels
Source: Plant Biotechnol J. 2017 Oct 13;16(4):877–89. doi: 10.1111/pbi.12835 (PMC5866951; doi:10.1111/pbi.12835)
Supplement: Supplementary file 2 — Figure S2 Gene ontology analysis in canola. [file PBI-16-877-s004.docx]

**Figure S2 – Gene ontology analysis in canola**


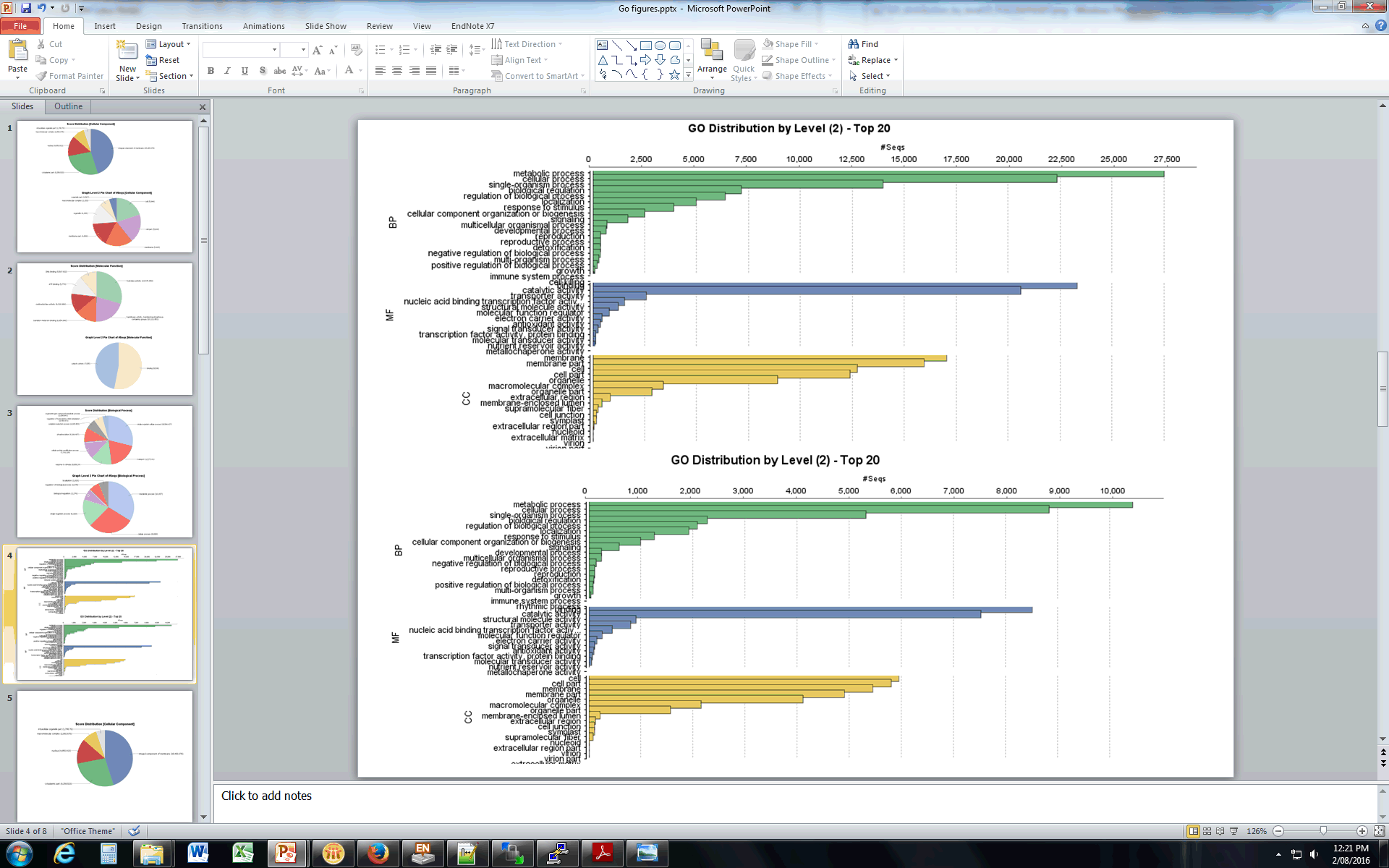


A

B

**Figure S2** Gene ontology classification based on BLAST to GO for (A) all of the genes identified within the canola genome with known locations and (B) all of the genes from the canola genome that have SNP loci identified using the GBS-t approach.

***Gene Class Analysis:***

To evaluate the spread of gene classes that are routinely sampled a comparison was made between all of the genes contained within the canola genome, as predicted using the CDS file, with the subset of genes that were identified with SNP loci through the GBS-t approach. A total of 88,428 genes are described within the CDS file with known locations and a total of 22,039 genes are identified within the set as the high quality variant set. A BLASTtoGO comparison was made between these two gene sets. Comparable rank ordering of gene classes were identified with a linear decrease in number identified in the SNP loci genes due to the lower number of genes in the data set.
